# A new prediction nomogram of non-sentinel lymph node metastasis in cT1-2 breast cancer patients with positive sentinel lymph nodes

**DOI:** 10.1038/s41598-024-60198-0

**Published:** 2024-04-26

**Authors:** Liu Yang, Xueyi Zhao, Lixian Yang, Yan Chang, Congbo Cao, Xiaolong Li, Quanle Wang, Zhenchuan Song

**Affiliations:** 1 Department of Breast Center, The Fourth Hospital of Hebei Medical University, Shijiazhuang, 050000 China; 2 Department of Breast Surgery, Xingtai People’s Hospital, Xingtai, 054000 China; 3 Department of Breast Surgery, Affiliated Hospital of Hebei Engineering University, Handan, 056000 China; 4 Department of Breast Surgery, The Fourth Hospital of Shijiazhuang, Shijiazhuang, 050000 China

**Keywords:** Breast cancer, Cancer models, Cancer therapy

## Abstract

We aimed to analyze the risk factors and construct a new nomogram to predict non-sentinel lymph node (NSLN) metastasis for cT1-2 breast cancer patients with positivity after sentinel lymph node biopsy (SLNB). A total of 830 breast cancer patients who underwent surgery between 2016 and 2021 at multi-center were included in the retrospective analysis. Patients were divided into training (*n* = 410), internal validation (*n* = 298), and external validation cohorts (*n* = 122) based on periods and centers. A nomogram-based prediction model for the risk of NSLN metastasis was constructed by incorporating independent predictors of NSLN metastasis identified through univariate and multivariate logistic regression analyses in the training cohort and then validated by validation cohorts. The multivariate logistic regression analysis revealed that the number of positive sentinel lymph nodes (SLNs) (*P* < 0.001), the proportion of positive SLNs (*P* = 0.029), lymph-vascular invasion (*P* = 0.029), perineural invasion (*P* = 0.023), and estrogen receptor (ER) status (*P* = 0.034) were independent risk factors for NSLN metastasis. The area under the receiver operating characteristics curve (AUC) value of this model was 0.730 (95% CI 0.676–0.785) for the training, 0.701 (95% CI 0.630–0.773) for internal validation, and 0.813 (95% CI 0.734–0.891) for external validation cohorts. Decision curve analysis also showed that the model could be effectively applied in clinical practice. The proposed nomogram estimated the likelihood of positive NSLNs and assisted the surgeon in deciding whether to perform further axillary lymph node dissection (ALND) and avoid non-essential ALND as well as postoperative complications.

## Introduction

Breast cancer is the most common malignancy in women worldwide and seriously threatens women's physical and mental health^1,2^. Axillary lymph node (ALN) metastasis is the earliest and most common metastatic pathway of breast cancer, and ALN status is one of the most important prognostic factors in breast cancer^3–5^. In recent years, sentinel lymph node biopsy (SLNB) has become a common method of assessing the axilla's status to achieve minimal trauma and the most efficient treatment^6^. SLNB can be a safe alternative to axillary lymph node dissection (ALND) when the sentinel lymph node (SLN) is negative^7,8^, and the management of the axilla when the SLN is positive is currently a hot topic of research^9^. According to the ACOSOG Z0011 trial, no axillary surgical treatment is recommended for patients with 1–2 positive SLNs who undergo breast-conserving surgery combined with postoperative radiotherapy. AMAROS and IBCSG23-01 offer evidence in favor of axillary management for patients with SLN micrometastases. However, the management of the axilla in patients undergoing mastectomy with 1–2 positive SLNs remains controversial.

SLN-positive patients inevitably undergo ALND, despite postoperative complications that can seriously affect the quality of life, such as arm/shoulder mobility restriction, lymphedema, numbness, and paresthesia^10^. Unfortunately, some studies have shown that 20–60% of SLN-positive patients have negative non-sentinel lymph nodes (NSLN) after ALND^11–13^. The feasibility of omitting ALND in patients with 1–2 SLN-positive has also been illustrated by the fact that some studies have shown no differences in disease-free survival (DFS) and overall survival (OS) between patients who undergo ALND and those who do not^14–16^. Therefore, it is urgent to develop a predictive model to assess the risk of NSLN metastasis and identify patients at low risk. However, how do surgeons determine if a patient with positive SLNs needs further axillary surgery?

Some conventional models could assess the NSLN metastasis in breast cancer patients who are SLN-positive, but the majority of these predictive models were developed using data from Western populations^17,18^. The effects of biomarkers, heredity, lifestyle, and socio-economic status on breast cancer vary among different races^19–22^. Additionally, there are fewer NSLN metastasis prediction models based on breast cancer in the Chinese population^23,24^. Several studies have assessed the predictive capacity of established models in predicting ALN metastasis in Chinese women breast cancer patients, but they found inconsistencies when compared to the original studies^7,25,26^. Xu Guo et al^27–29^. developed a prediction model that combined axillary ultrasonography (AUS) with deep learning radionics (DLR). The model could be conveniently applied in primary hospitals. However, its predictive capacity was low when imaging was negative due to inherent ultrasound defects. Ke Xiang et al^14^. developed a model to predict the risk of NSLN metastasis, but they used a limited number of cases and lacked external validation.

Therefore, predictors and prediction models for NSLN metastasis in cT1-2 breast cancer patients need further research. In this study, we developed a new nomogram to assess the risk of NSLN metastasis, enabling surgeons to avoid unnecessary ALND by screening patients with a low risk of NSLN metastasis.

## Results

### Clinical and pathological characteristics

**Figure 1:**
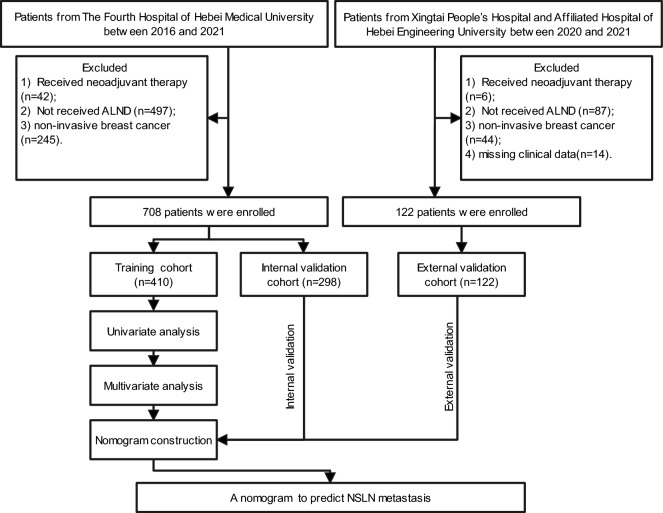
Flowchart of patient selection and nomogram construction. ALND: axillary lymph node dissection.

A total of 1,765 cT1-2 breast cancer patients with SLN metastases during the review period and 935 of these cases were excluded according to the exclusion criteria (48 cases for received neoadjuvant therapy, 584 for not receiving ALND, 289 for non-invasive breast cancer, and 14 for missing clinical data). Finally, 830 cT1-2 breast cancer patients with positive SLNs were enrolled and were assigned to the training cohort (*n* = 410), internal validation cohort (*n* = 298), and external validation cohort (*n* = 122) according to the periods and centers. Of the 830 patients enrolled, 525 patients (63.3%) had one positive SLN, 206 (24.8%) had two positive SLNs, and 99 (11.9%) had three or more positive SLNs. The average age in the training, internal validation, and external validation cohorts was 51.0 ± 11.0, 50.7 ± 10.8, and 52.1 ± 9.9 years, respectively. The mean tumor size was 1.85 ± 0.95, 2.00 ± 0.87, and 1.97 ± 0.95 cm in the corresponding cohorts. The number of positive SLNs was 1.56 ± 0.89, 1.52 ± 0.82, and 1.44 ± 0.75, and the positive rate of NSLN metastasis was 26.8%, 24.2%, and 38.5% in the training, internal validation, and external validation cohorts. Descriptive characteristics of the population are presented in Table 1 and Fig. 1.

**Table 1:** Clinical and pathological characteristics of patients in the training, internal validation, and external validation cohorts.

Characteristics	Training cohort n (%)	Internal validation cohort n (%)	External validation cohort n (%)
Cases	410 (100.0%)	298 (100.0%)	122 (100.0%)
Age			
≤ 50	116 (28.3%)	112 (37.6%)	61 (50.0%)
> 50	294 (71.7%)	186 (62.4%)	61 (50.0%)
Menstrual status			
Premenopausal	188 (45.9%)	168 (56.4%)	67 (54.9%)
Postmenopausal	222 (54.1%)	130 (43.6%)	55 (45.1%)
Surgery			
Mastectomy	361 (88.0%)	243 (81.5%)	103 (84.4%)
Breast-conserving	49 (12.0%)	55 (18.5%)	19 (15.6%)
Tumor size			
≤ 2 cm	258 (62.9%)	159 (53.4%)	99 (81.1%)
2-5 cm	152 (37.1%)	139 (46.6%)	23 (18.9%)
Number of positive SLNs			
≤ 2	358 (87.3%)	265 (88.9%)	108 (88.5%)
> 2	52 (12.7%)	33 (11.1%)	14 (11.5%)
Proportion of positive SLNs			
≤ 0.5	307 (74.9%)	221 (74.2%)	79 (64.8%)
> 0.5	103 (25.1%)	77 (25.8%)	43 (35.2%)
Number of positive NSLNs			
0	300 (73.2%)	226 (75.8%)	75 (61.5%)
1–3	80 (19.5%)	54 (18.1%)	35 (28.7%)
≥ 4	30 (7.3%)	18 (6.1%)	12 (9.8%)
Lymph-vascular invasion			
Negative	331 (80.7%)	231 (77.5%)	93 (76.2%)
Positive	79 (19.3%)	67 (22.5%)	29 (23.8%)
Perineural invasion			
Negative	342 (83.4%)	274 (91.9%)	96 (78.7%)
Positive	68 (16.6%)	24 (8.1%)	26 (21.3%)
Histological grade			
I	17 (4.2%)	30 (10.1%)	5 (4.1%)
II	299 (72.9%)	203 (68.1%)	99 (81.1%)
III	94 (22.9%)	65 (21.8%)	18 (14.8%)
ER			
Negative	36 (8.8%)	44 (14.8%)	24 (19.7%)
Positive	374 (91.2%)	254 (85.2%)	98 (80.3%)
PR			
Negative	57 (13.9%)	50 (16.8%)	32 (26.2%)
Positive	353 (86.1%)	248 (83.2%)	90 (73.8%)
HER-2			
Negative	314 (76.6%)	233 (78.2%)	100 (82.0%)
Positive	96 (23.4%)	65 (21.8%)	22 (18.0%)
Ki-67			
≤ 20%	192 (46.8%)	152 (51.0%)	49 (40.2%)
> 20%	218 (53.2%)	146 (49.0%)	73 (59.8%)
Molecular subtype			
Luminal A	182 (44.4%)	140 (47.0%)	55 (45.1%)
Luminal B	116 (28.3%)	75 (25.2%)	27 (22.1%)
HER-2 enriched	96 (23.4%)	65 (21.8%)	31 (25.4%)
Triple-negative	16 (3.9%)	18 (6.0%)	9 (7.4%)
Chemotherapy			
Yes	372 (90.7%)	265 (88.9%)	112 (91.8%)
No	38 (9.3%)	33 (11.1%)	10 (8.2)
Radiotherapy			
Yes	232 (56.6%)	179 (60.1%)	59 (48.4%)
No	178 (43.4%)	119 (39.9%)	63 (51.6%)
Endocrinotherapy			
Yes	338 (82.5%)	268 (89.9%)	104 (85.2%)
No	72 (17.5%)	30 (10.1%)	18 (14.8%)

SLNs: sentinel lymph nodes; NSLNs: non-sentinel lymph nodes; ER: estrogen receptors; PR: progesterone receptors; HER-2: human epidermal growth factor receptor 2.

### Independent predictors for NSLN metastasis

The univariate analysis based on clinicopathologic information was conducted to explore the potential predictors of NSLN metastasis in the training cohort. As shown in Table 2, the number of positive SLNs (*P* < 0.001), the proportion of positive SLNs (*P* < 0.001), lymph-vascular invasion (*P* = 0.001), perineural invasion (*P* = 0.003), and ER status (*P* = 0.029), were detected to be significantly associated with NSLN metastasis. These five variables were further incorporated into multivariate logistic regression analyses. The results showed that the number of positive SLNs (*P* < 0.001; OR: 1.801; 95% CI: 1.295–2.506), the proportion of positive SLNs (*P* = 0.029; odds ratio (OR): 3.671; 95% confidence intervals (CI): 1.139–11.832), lymph-vascular invasion (*P* = 0.029; OR: 1.790; 95% CI: 1.063–3.016), perineural invasion (*P* = 0.023; OR: 1.984; 95% CI: 1.098–3.583), and estrogen receptor (ER) status (*P* = 0.034; OR: 3.164; 95% CI: 1.092–9.165) were identified as independent risk factors for NSLN metastasis. Therefore, factors such as the positive number of SLNs > 2, the proportion of positive SLNs > 50%, lymph-vascular invasion, perineural invasion, and ER-positive were considered risk factors for NSLN metastasis.

**Table 2:** Univariate and multivariate analysis of the characteristics in training cohort.

Characteristics	Training cohort		Univariate analysis		Multivariate analysis	
	NSLN ( +)	NSLN(-)	χ^2^	*P*	OR (95%CI)	*P*
	*n* (%)	*n* (%)				
Cases	110	300				
Age			0.075	0.784		
≤ 50	33(30.0%)	83(27.7%)				
> 50	77(70.0%)	217(72.3%)				
Menstrual status			0.843	0.426		
Premenopausal	54(49.1%)	134(44.7%)				
Postmenopausal	56(50.9%)	166(55.3%)				
Tumor size			1.451	0.228		
≤ 2 cm	64(58.2%)	194(64.7%)				
2-5 cm	46(41.8%)	106(35.3%)				
Number of positive SLNs			36.547	** < 0.001**	1.801(1.295–2.506)	**<0.001**
≤ 2	78(70.9%)	280(93.3%)				
> 2	32(29.1%)	20(6.7%)				
Proportion of positive SLNs			24.771	**<0.001**	3.671(1.139–11.832)	**0.029**
≤ 0.5	63(57.3%)	244(81.3%)				
> 0.5	47(42.7%)	56(18.7%)				
Lymph-vascular invasion			4.865	**0.001**	1.790(1.063–3.016)	**0.029**
Negative	81(73.6%)	250(83.3%)				
Positive	29(26.4%)	50(16.7%)				
Perineural invasion			8.548	**0.003**	1.984(1.098–3.583)	**0.023**
Negative	82(74.5%)	260(86.7%)				
Positive	28(25.5%)	40(13.3%)				
Histological grade			3.858	0.145		
I	3(2.7%)	14(4.7%)				
II	88(80%)	211(70.3%)				
III	19(17.3%)	75(25%)				
ER			4.967	**0.029**	3.164(1.092–9.165)	**0.034**
Negative	4(3.6%)	32(10.7%)				
Positive	106(96.4%)	268(89.3%)				
PR			2.908	0.088		
Negative	10(9.1%)	47(15.7%)				
Positive	100(90.9%)	253(84.3%)				
HER-2			0.214	0.644		
Negative	86(80.9%)	228(78.3%)				
Positive	24(19.1%)	72(21.7%)				
Ki-67	1.503	0.22	1.503	0.220		
≤ 20%	57(51.8%)	135(45%)				
> 20%	53(48.2%)	165(55%)				
Molecular subtype						
Luminal A	59(53.6%)	123(41.0%)				
Luminal B	18(16.4%)	98(32.7%)	0.38	0.001	0.391 (0.221–0.702)	0.002
HER-2 enriched	31(28.2%)	14(4.7%)	0.30	0.117	1.484 (0.172–12.693)	0.723
Triple-negative	2(1.8%)	65(21.6%)	0.99	0.983	1.243 (0.711–2.152)	0.448
Chemotherapy			0.006	0.940		
Yes	100(90.9%)	272(90.7%)				
No	10(9.1%)	28(9.3%)				
Radiotherapy			3.042	0.081		
Yes	70(63.6%)	162(54.0%)				
No	40(36.4%)	138(46.0%)				
Endocrinotherapy			1.599	0.206		
Yes	95(86.4%)	243(81.0%)				
No	15(13.6%)	57(19.0%)				

NSLN ( +): positive non-sentinel lymph nodes; NSLN (-): negative non-sentinel lymph nodes; OR: odds ratio; CI: confidence interval; SLNs: sentinel lymph nodes.

Bold values representing statistical significance.

χ^2^: chi-squared test.

### Nomogram construction

**Figure 2:**
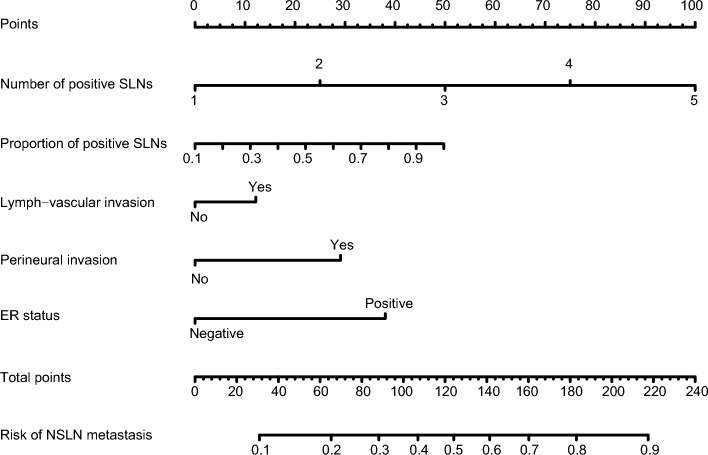
Nomogram predicting the probability of NSLN metastasis. The five variables were located in rows 2–6 of the nomogram. The corresponding scores were obtained by inputting each of the patient's characteristics into the nomogram and drawing an upward line to row 1 (Points). The scores were then summed and a line was drawn downwards to row 7 (Total points) to obtain the total score. Put the total score in a downward line to row 8 (Risk of NSLN metastasis) to determine the risk of NSLN metastasis.

Based on the five risk factors obtained from the multivariate analysis in the training cohort, the model was presented as a visible nomogram. As shown in Fig. 2, the number of positive SLNs had the greatest effect on NSLN metastasis with a maximum score of 100 points. Next in influence was the proportion of positive SLNs with a maximum score of 50 points. The effect of ER status has a maximum score of 38 points. The effects of perineural invasion and lymph-vascular invasion were not negligible with 29 and 12 points, respectively.

### Model validation

**Figure 3:**
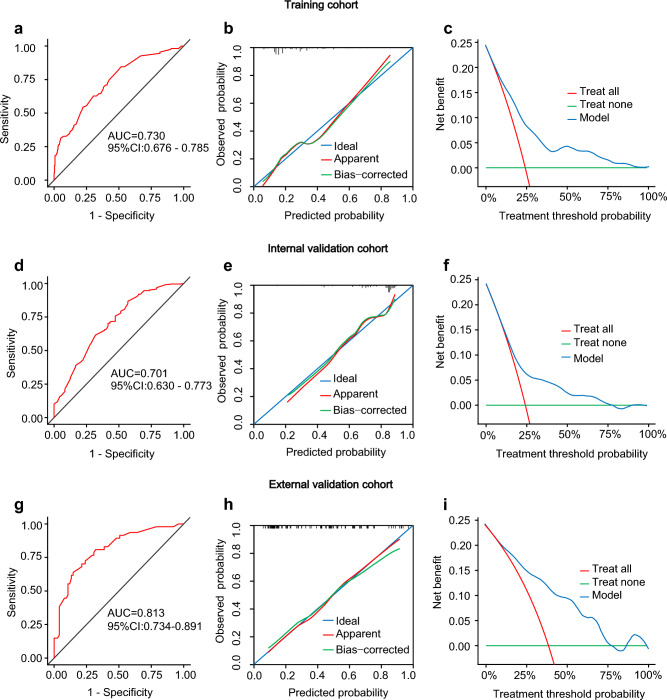
Nomogram verification for training cohort **(a–c)**, internal validation cohort **(d–f)**, and external validation cohort **(g-i)**. a, d, g: ROC curves. b, e, h: calibration curves. The "ideal" line as the reference standard. The "apparent" line shows the agreement between the observed and predicted probabilities. The "bias-corrected" line shows the agreement between the corrected predicted and the observed probabilities. A closer proximity of the apparent or bias-corrected line to the ideal line indicates better consistency between predicted values and actual values. c, f, i: DCA curves. The "treat all" line represents the assumption that all non-sentinel lymph nodes of the patients were positive. The "treat none" line represents the assumption that none non-sentinel lymph nodes of the patients were positive. The "model" line represents the nomogram. The DCA curve lies above the "none" and "all" baselines in the threshold probability range of 0.1 to 0.7, indicating acceptable model performance in this range. ROC: receiver operating characteristics; AUC: areas under the ROC curve; CI: confidence interval; DCA: decision curve analysis.

To evaluate the predictive capacity of the nomogram model for the NSLN metastasis risk, we used the receiver operating characteristic (ROC) curve. As shown in Fig. 3a,d,g, the area under the curve (AUC) was 0.730 (95%CI: 0.676–0.785), 0.701 (95%CI: 0.630–0.773), and 0.813 (95% CI: 0.734–0.891) in the training, internal validation, and external validation cohorts. The calibration curve and the decision curve analysis (DCA) were plotted to assess the nomogram's effectiveness. The calibration curves (Fig. 3b,e,h) illustrated a strong agreement between the predicted and observed values across all three cohorts. The DCA demonstrated more benefits within the probability range of 10%-70%, as shown in Fig. 3c,f,i. Overall, the nomogram shows a good ability to discriminate and calibrate.

Among the training cohort, at a cutoff of 14% for the risk of NSLN metastasis (with total points of 50), there were 106 cases reported as "negative", of which 98 cases were true negatives based on the results of ALND and 8 cases that were false negatives, and 304 reported as positive (102 true positives and 202 false positives). The Youden Index ranges from − 1 to 1, and a value closer to 1 indicates a better discriminatory ability of the test. The cutoff value of 26% is determined based on the maximum Youden index. The sensitivity, specificity, false-negative rate, false-positive rate, and negative predictive value of different cutoffs are shown in Table 3. The false-negative rate below 10% is generally considered acceptable. Considering the clinical application, we set the cut-off value for the nomogram at 14%, which means that if the risk of NSLN metastasis is less than 14% (50 points in total), it is recommended to omit ALND.

**Table 3:** Diagnostic performance of the predictive nomogram at different cutoff values in the training, internal validation, and external validation cohorts.

	Cutoff	Sensitivity	Specificity	FNR	FPR	NPV
Training cohort	10%	98.20%	7.70%	1.80%	92.3%	92.00%
	14%	92.70%	32.70%	7.30%	67.3%	92.50%
	26%	62.70%	67.30%	37.30%	32.7%	83.10%
Internal validation cohort	10%	94.40%	11.50%	5.60%	88.5%	86.70%
	14%	90.30%	34.50%	9.70%	65.5%	91.80%
	26%	54.20%	69.50%	45.80%	30.5%	82.60%
External validation cohort	10%	97.90%	13.30%	2.10%	86.7%	90.90%
	14%	93.60%	44.00%	6.40%	56%	91.70%
	26%	66.00%	73.30%	34.00%	26.7%	77.50%

FNR: False-negative rate; FPR: False positive rate; NPV: Negative predictive value.

Cutoff: When the risk of NSLN metastasis is lower than this value, exemption of ALND is recommended.

## Discussion

The concept of breast cancer treatment is gradually shifting towards precision and individualized treatment, and how to maximize the benefits of treatment while minimizing the trauma is a goal that surgeons have been pursuing^30^. The Z0011 trial results have influenced treatment decisions in breast cancer by allowing SLN-positive patients who underwent breast-conserving surgery combined with postoperative radiotherapy to omit ALND^31^. In our study, approximately 14.8% (123/830) of patients received breast-conserving surgery. Although our study focused on patients who may not be fully matched to the Z0011 trial, it provided a more representative perspective on breast cancer in the Chinese population. Notably, nearly 60% of breast cancer patients with positive SLNs in some studies did not show NSLN metastases in further ALND. Within our study population, 72.4% had no detectable NSLN metastases. To identify SLN-positive and NSLN-negative patients, we analyzed the clinical and pathological characteristics of SLN-positive but NSLN-negative patients, incorporated meaningful factors for further analysis, and constructed a visual nomogram.

Through univariate analysis and multivariate logistic regression analysis in the training cohort, we have identified five parameters that are associated with NSLN metastasis: the number of positive SLNs, the proportion of positive SLNs, lymph-vascular invasion, perineural invasion, and ER status. Consistent with previous studies, a high number of positive SLNs and a high proportion of positive SLNs were associated with NSLN metastases^7,15,32^. An increased number of SLN metastases usually indicates a higher likelihood of lymph node involvement, which predicts an increased risk of NSLN metastases. However, the number of SLNs detected during surgery is influenced by the experience of the surgeon, the usage of the tracer, and anatomical differences in the patient. A higher proportion of positive SLNs may lead to an overestimation of the risk of NSLN metastasis if too few SLNs are detected during surgery. To reduce the bias resulting from the reasons mentioned above, the majority (797, 96.0%) of SLNs detected during surgery in our study were two or more. To further explore the occurrence of NSLN metastasis in patients with 1–2 SLN metastases, we divided the training cohort of patients with 1 or 2 SLN metastases into the 1-SLN-positive group and the 2-SLN-positive group. We found that 2-SLN-positive patients had a higher NSLN metastasis rate (31.7% vs. 17.7%, *P* = 0.004). Positive lymph-vascular invasion and perineural invasion indicate a more aggressive tumor with greater metastatic potential. Consistent with previous research^33–35^, our multivariable analyses showed that lymph-vascular invasion and perineural invasion were independent risks of NSLN metastasis. Currently, the association between ER status and NSLN metastasis is contentious^36^. Several studies have shown a negative association between ER status and NSLN metastasis^14,35,37,38^. In our investigation, ER positivity was a risk factor for NSLN metastasis, which is consistent with the findings of the study conducted by Alsumai et al^34,39,40^.

Furthermore, there was no significant connection between HER-2 status and NSLN metastasis found in our study (*P* = 0.644), which differs from the findings of Zhao et al^35,38,40,41^. An increased Ki-67 index is usually accompanied by a highly metastatic state^42^. However, our study did not find a relationship between Ki-67 and NSLN metastasis, which differs from the findings of the study conducted by Augusto Pereira et al^3,43^. Inconsistent conclusions may be attributed to ethnic differences and require further research.

Several models have been proposed to predict the presence of NSLN metastasis in breast cancer patients. The MSKCC nomogram contains eight predictors with an AUC of 0.711–0.761 in the Chinese population^7,10,13,25^. Including fewer parameters in the nomogram than in the MSKCC nomogram would not only reduce the interactions caused by the inclusion of too many factors but also provide greater practicality for real-world applications. Our nomogram was composed of five variables which included the number of positive SLNs, the proportion of positive SLNs, lymph-vascular invasion, perineural invasion, and ER status. The established nomogram had an excellent performance in external validation, suggesting its wide applicability. For patients with a low risk of NSLN metastasis, omission of ALND may be considered. Moreover, a false-negative rate of less than 10% is acceptable clinically^25^. When the predicted cutoff value in our nomogram was 14%, the sensitivity, specificity, false-negative rate, and false-positive rate were 92.7%, 32.7%, 7.3%, and 67.3%. Therefore, considering the clinical application and false-negative rate, the cutoff value of the nomogram is finally set to 14%, which means that if the NSLN metastatic rate was less than 14% according to our prediction nomogram, the exemption of ALND would be recommended. For example, a 65-year-old breast cancer patient who is ER-positive with negative lymph-vascular invasion and perineural invasion. In this case, five SLNs were detected, with one SLN positivity, the overall score was 44, corresponding to a 13% probability of NSLN metastasis. This patient falls into the low-risk group and may be a candidate for omitting ALND. The studies by Gao et al. found that the survival benefit from ALND was not significant in mastectomy patients with 1–2 positive SLN. These studies also provide evidence supporting the omission of ALND^16,44,45^.

The present study still has some limitations. First, this study is retrospective, and larger prospective studies are needed to validate the model. Second, the external cohort sample is limited and requires a multi-center prospective study to increase the number of cases to improve the accuracy and representativeness of the prediction model.

## Conclusions

In conclusion, we found that the number of positive SLNs, the proportion of positive SLNs, lymph-vascular invasion, perineural invasion, and ER status were independent risk factors for NSLN metastasis and utilized these factors to develop a nomogram. The nomogram showed good predictive performance. To maximize patient benefit while minimizing damage to the organism, surgeons can use this nomogram to identify patients with a low risk of NSLN metastasis and omit further ALND. Our nomogram has clinical applicability.

## Materials and methods

### Patients

The clinical data of patients with cT1-2 breast cancer who underwent surgery from January 2016 to December 2021 at multi-center was retrospectively analyzed. All patients were classified as cN0 (negative axillary ultrasound or clinical examination). Inclusion criteria: (1) 18 years of age or older; (2) Paraffin pathology diagnosis of invasive breast cancer (3) One or more SLN macrometastases; (4) Successful SLNB and ALND; (5) The preoperative clinical diagnosis was T1-2, N0 according to the 8th American Joint Committee on Cancer (AJCC). Exclusion criteria: (1) Presence of other malignant tumors; (2) Metastatic breast cancer; (3) Having undergone only SLNB without ALND; (4) Treated with neoadjuvant chemotherapy or radiotherapy; (5) Missing clinical data. Finally, a total of 830 patients were included for further analysis.

Training cohort: 410 patients treated at The Fourth Hospital of Hebei Medical University between 2016 and 2019.

Internal validation cohort: 298 patients treated at The Fourth Hospital of Hebei Medical University between 2020 and 2021.

External validation cohort: 122 patients treated at Xingtai People's Hospital (*n* = 94) and the Affiliated Hospital of Hebei Engineering University (*n* = 28) between 2020 and 2021.

### Ethics approval and consent to participate

This retrospective study was approved by The Ethics Committee of The Fourth Hospital of Hebei Medical University (2022KY054). According to the requirements of the ethics committee, local legislation, and institutional requirements, informed consent was waived by The Ethics Committee of The Fourth Hospital of Hebei Medical University because of the retrospective nature of our study. The study was performed in compliance with the Declaration.

### Data collection

Data was collected from eligible patients’ records, including age, type of surgery, tumor size, the number of positive SLNs, proportion of positive SLNs, number of positive NSLNs, lymph-vascular invasion, perineural invasion, histological grade, the status of estrogen receptor (ER), progesterone receptor (PR), human epidermal growth factor-2 (HER-2), and Ki-67.

### Diagnostic criteria

The status of ER, PR, and HER-2 was identified through immunohistochemistry (IHC). According to the 2020 American Society of Clinical Oncology/College of American Pathologists Guideline (2020 ASCO/CAP), tumor nuclei staining of ≥ 1% was defined as positive for ER or PR, and nuclei staining of < 1% was defined as negative^46^. HER-2 status was defined according to the results of IHC: 0 or 1 + were considered HER-2 negative, while 3 + was considered HER-2 positive. In cases of 2 + , additional fluorescence in situ hybridization (FISH) was required to determine whether the HER-2 gene was amplified or not. HER-2 status was considered negative unless the HER-2 gene was amplified; otherwise, it was considered positive^47,48^.

### SLNB and ALND procedures

Patients who tested SLN-positive would undergo further ALND. In the cases where the intraoperative frozen section (FS) or touch imprint cytology (TIC) showed a negative SLN but the postoperative paraffin pathology revealed a positive SLN, a secondary ALND was performed^25^.

### Statistical analysis

Statistical analysis was conducted using SPSS 22.0. Continuous variables were presented as mean ± standard deviation, while categorical variables were presented as proportions. The chi-squared test or Fisher exact test was used to compare categorical variables between groups. To identify risk factors affecting NSLN metastasis, a univariate analysis was performed in the training cohort. Factors found to be statistically significant underwent multivariate logistic regression analysis. The results were then presented as OR along with their corresponding 95% CI. All tests were two-sided, and *P* < 0.05 indicated a statistically significant difference.

A predictive model for the risk of NSLN metastasis was constructed using R software (version 4.2.2) along with MSTATA software (www.mstata.com). A nomogram model was established according to the screened independent risk factors, and validated by the internal and external validation cohorts. The ROC was plotted to calculate the AUC and evaluate the predictive power of the nomogram model. The AUC over 0.7 indicates that the nomogram provides a reasonable estimation. A calibration curve and DCA were used to evaluate the performance of the model.

## Data Availability

The datasets generated and analyzed during the current study are available from the corresponding author upon reasonable request.
